# Clinicopathological Profile of Primary Extra Nodal Lymphoma from a Tertiary Care Center in South India

**DOI:** 10.30699/IJP.2024.2018132.3229

**Published:** 2024-01-29

**Authors:** Nischitha N Suvarna, Vidya Monappa

**Affiliations:** *Department of Pathology, Kasturba Medical College Manipal, Manipal Academy of Higher Education, Manipal, Karnataka, India*

**Keywords:** Extra nodal lymphoma, pENL, Primary ENL

## Abstract

**Background & Objective:**

Primary extranodal lymphoma (pENL) is a malignant lymphoid neoplasm that presents with the main bulk of disease at an extranodal site. The incidence of primary pENL has risen sharply in recent years due to the advent of better diagnostic modalities. Diagnosing pENL can be challenging due to its morphological overlap with other tumors native to the site of origin. This study aims to establish the anatomic distribution, clinical presentations, possible etiologic correlations, and histological subtypes of pENL in a tertiary care center located in South India.

**Methods:**

This is a retrospective study of 109 patients with pENL (69 males, 40 females, M: F = 1.7:1) over 5 years (October 2012 to September 2017). The tumors were reclassified according to WHO classiﬁcation of Haematolymphoid tumors, 5th edition, 2022.

**Results:**

pENL constituted 109/481 cases (22.6%) of all NHL cases, with the highest incidence in 7th decade. The gastrointestinal tract (39%) was the predominant site involved, followed by head and neck (26%). Diffuse large B cell lymphoma (DLBCL) was the most common histomorphological variant followed by Follicular lymphoma (FL). The majority of the patients were immunocompetent (89%) and presented with stage IV disease (31.1%) at diagnosis.

**Conclusion:**

This study presents an overview of the diverse distribution patterns of both common and rare pENL within a tertiary care center. The accurate diagnosis of pENL necessitates the elimination of secondary extranodal involvement. It is important to note that the accurate diagnosis of pENL requires careful evaluation and exclusion of other possible causes.

## Introduction

Primary extranodal lymphomas (pENL) account for about 25% - 40% of all non-Hodgkin lymphomas (NHL) (1,2). pENL presents with the bulk of disease at an extranodal site, usually necessitating the direction of treatment primarily to that site (3). The origin of these tumors is widespread and can be seen even in sites that normally do not contain lymphoid tissue. 

pENL can be diagnosed as per Dawson’s criteria (4): 1) Lack of superficial palpable lymph nodes; 2) Plain Chest X-ray shows absence of mediastinal lymphadenopathy; 3) Major bulk in an extranodal site; 4) Nodal involvement in the area surrounding the primary site; 5) Normal limit of white blood cell (WBC) count. Secondary involvement of extranodal sites as a result of disease progression or advanced stage does not qualify as pENL. Historically, the classification of pENL was similar to that of nodal lymphomas. The first attempt to classify them was made in the Revised European-American Lymphoid Neoplasms (REAL) classification (1994) (5). The 2016 classification divided lymphoid neoplasms into precursor, mature lymphoid, histiocytic, and dendritic neoplasms (6). This has been further refined in the 2022 5^th^ edition of WHO, which now incorporates essential and desirable diagnostic criteria for each entity and offers a more integrated approach to diagnosis with morphologic, immunophenotypic, molecular, and cytogenetic details (7). 

Diagnosis of ENL can be challenging to the pathologist, due to the histologic mimics, molecular and genetic variation, and diverse clinical presentations. Consequent to the AIDS epidemic, multifactorial risk factors like EBV, HCV, HHV8, Helicobacter pylori, *Campylobacter jejuni*, *Chlamydia psittaci*, Tuberculosis, autoimmune diseases, Hashimoto’s thyroiditis, celiac disease, inflammatory bowel disease, there has been an upsurge in pENL cases. (3,8). The site of involvement of ENL reflects the homing characteristics of the lymphoid population from which they arise (9). The clinical signs and symptoms at presentation along with the outcomes depend largely on the organ involved. Due to the geographic and demographic variations, it presents a frequent challenge to diagnose, classify, and provide appropriate treatment to pENL patients. There exist wide-ranging differences in the incidence of pENL globally, with very little information from developing countries. Hence, this study in South India aims to determine the distribution, presentations, histological subtypes, and sites involved in pENL. The findings will aid in the development of effective diagnostic and treatment strategies and enhance patient care. 

The study’s focus on a tertiary care center in South India provides region-specific information on pENL. It explores demographics, clinical presentations, histological subtypes, and sites involved over 5 years. Staging, immunophenotyping, and long-term follow-up remain crucial for a comprehensive understanding of pENL.

## Material and Methods

This was a 5-year retrospective cohort study of patients diagnosed with pENL in our tertiary care referral center over 5 years (October 2012 to September 2017). The patient’s data regarding age, sex, demography, occupation, detailed clinical history, physical examination, routine hematological examination (CBC, ESR, Peripheral smear, Bone marrow biopsy), biochemical parameters (LDH), microbiology status (HIV, HBV, HCV, EBV, H. pylori), radiological findings (wherever available), diagnosis, staging, response to therapy in already diagnosed cases of pENL were retrieved from hospital records. 

To provide a comprehensive overview, we analyzed the gross features like the size, number, appearance, and regional lymph node status of the resected specimen recovered from archival data. The Haematoxylin & eosin-stained slides were reviewed for microscopic features like cell size, nuclear details, mitosis, necrosis, and cellular arrangement. Immunohistochemistry slides were reviewed and included a varied, case-specific panel of markers: CD45, CD20, CD3, CD10, BCL2, BCL6, CD5, Cyclin D1, CD30, MUM-1, CD79a, CD23, CD21, CD8, CD4, CD56, CK, GFAP, Ki67, CD117, CD99, TdT, ALK, EMA, Synaptophysin and Chromogranin.

All cases were reclassiﬁed based upon morphologic and immunophenotypic criteria according to WHO 2022 classiﬁcation. The staging was done according to the Ann Arbor classification modified by Musshoff (10). The hospital records of the patients diagnosed with pENL were followed up for a maximum duration as documented in the medical records. 

Exclusion criteria: Secondary extranodal involvement, inadequate clinical data, and non-availability of blocks. Primary cutaneous lymphomas were excluded as they feature a diverse category and have a different classification system.

## Results

**Fig. 1:**
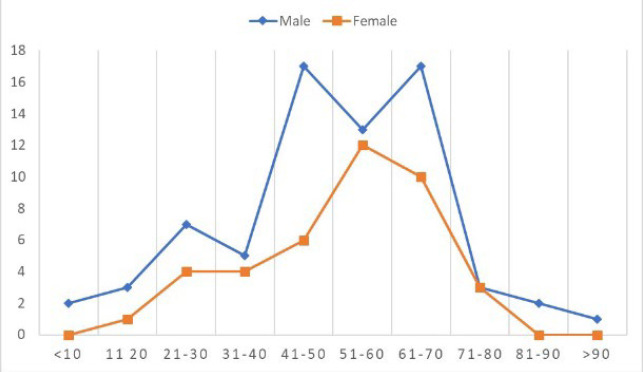
Age and Sex distribution of pENL

This is a retrospective cohort study spanning 5 years from October 2012 to September 2017. During this period 583 cases of lymphoma were diagnosed in our centre, of which 481 were NHL and 102 were HL cases. These included both nodal and extranodal NHL and HL. pENL constituted 109/481 cases (22.6%) of all NHL diagnosed during this period. There were 69 male patients and 40 female patients with an M: F ratio of 1.7:1. The age at diagnosis ranged from 5-93 years with a mean age of 50 years. The peak incidence of pENL was during the 7th decade contributing to 50.45% of cases as is depicted in Figure 1. 

Abdominal pain was the most common presenting symptom (37%), followed by vomiting (12%) and abdominal mass (12%). B symptoms (fever, weight loss) were seen in 20% of pENL cases. Underlying risk factors such as Hashimoto’s thyroiditis were seen in 2/109 (1.83%), HBV in 1/109 (0.9%), and EBV in 1/109 (0.9%) of cases. 12 (11%) cases showed bone marrow infiltration. The laboratory findings in this study showed elevated LDH in 39 cases (36%) and high ESR was seen in 62 cases (56.8%) of pENL.

**Fig. 2:**
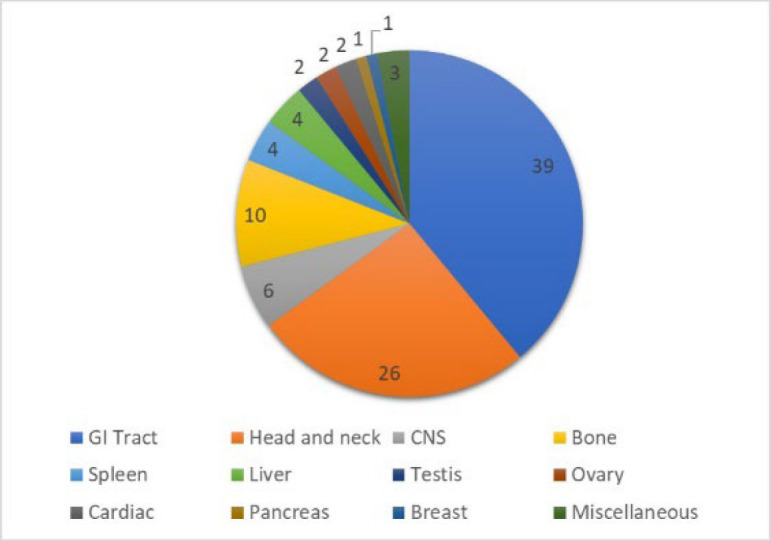
Site-wise distribution of pENL

The site distribution of pENL is depicted in Figure 2. The gastrointestinal tract (39%) was the most common site involved followed by head and neck (26%) and bone (10%). In the GIT, small intestine was the most common site involved (n=15, 35%). 

**Fig. 3:**
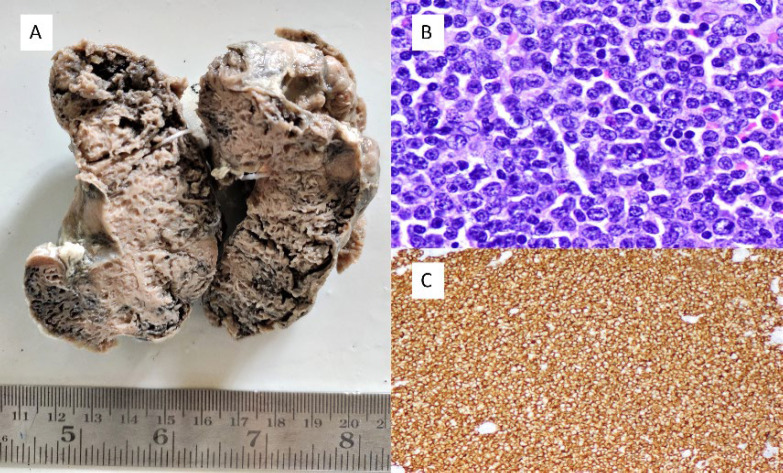
Primary Thyroid Diffuse large B cell lymphoma - (A) Gross shows an enlarged thyroid with multiple cystic and grey tan areas, (B) H and E x200, Large neoplastic cells diffusely infiltrating the thyroid follicles (C) CD20 positive (CD20, x200)

**Fig.4:**
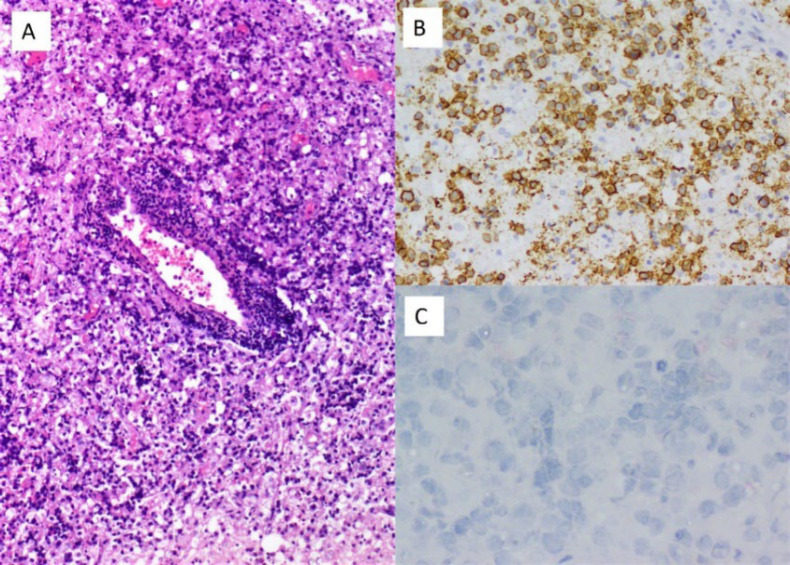
Primary CNS Diffuse large B cell lymphoma - (A) H and E x100, Perivascular aggregates of large lymphoid cells (B) CD20 positive (CD20, x200), (C) CD10 negative (CD10, x400).

**Fig. 5:**
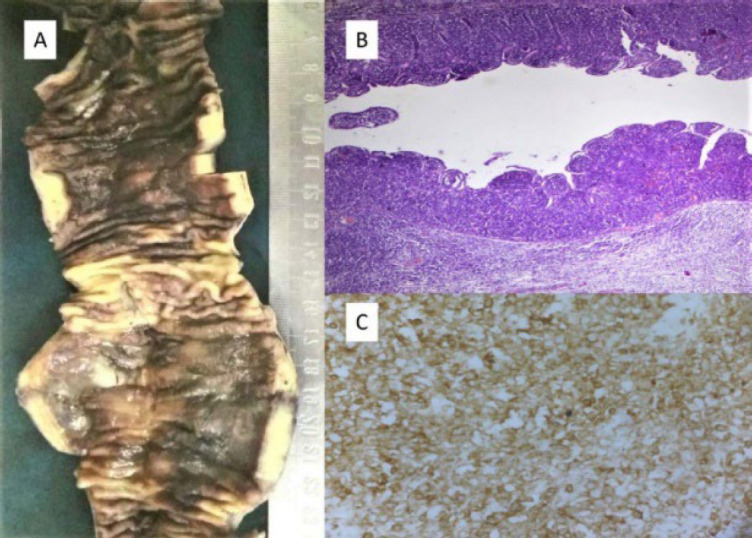
Enteropathy associated T cell lymphoma - (A) Gross shows diffuse thickening of the intestinal wall with ulcerations, (B) H and E, x20, Flattened and broadened villi with intraepithelial lymphocytes and diffuse lymphoid infiltrate, (C) CD3 positive (CD3, x20).

The majority of the pENL cases were of B cell phenotype (92/109, 84.4%) with only 8.25% (9 cases) being of T cell phenotype. 8/109 cases (7.3%) were not subjected to immunophenotyping and thus were classified as NHL NOS. DLBCL (55.1%) was the commonest histomorphological variant of pENL (Figures 3 and 4 ) followed by FL (9.17%) and MALT lymphoma (8.25%). There were 3 cases of T cell lymphomas, of which 2 were in the GIT (Figure 5). Table 1 highlights the site-wise distribution of the various histological subtypes of pENL as per the WHO 2022 classification. Table 2 compares the histological variants of pENL described.

**Table 1:** Site-wise distribution of histological subtype in pENL (n=109) as per the WHO 2022 classification:

SITE	DLBCL (n=58, 55.1%)	MALT (n=9,8.25%)	FL (n=10, 9.17%)	MCL (n=2, 1.8%)	T cell lymphoma (n=2, 1.8%)	EATL (n=1, 0.9%)	PBL (n=32.75%)	BL (n=2, 1.8%)	SMZL (n=1, 0.9%)	PTCL, NOS (n=2, 1.8%)	ALCL (n=2, 1.8%)	Primary CNS DLBCL (n=6, 5.5%)	NK cell (n=1, 0.9%)	TLL (n=1, 0.9%)	BLL (n=1, 0.9%)	NHL NOS (n=8, 7.3%)
GIT (n=43, 39%)	25	7	1	2	1	1	1	1								4
Stomach	6	3			1											2
Small intestine	9	3				1		1								1
Ileocecal	4	1		1												
Colon	5		1	1			1									1
Head and Neck (n=29, 26%)																
Nasopharynx	5		2		1								1			1
Tonsil	6		1							1						
Nasal cavity	1						1			1						
Thyroid	4	1														
Eye		1	1				1									
Bone (n=11, 10%)	7		2								1				1	
CNS (n=7, 6%)												6				1
Spleen (n=4, 4%)	2		1						1							
Liver (n=4, 4%)	1		1					1								1
Testis (n=2, 2%)	2															
Ovary (n=2, 2%)	2															
Cardiac (n=2, 2%)	2															
Breast (n=1,1%)														1		
Pancreas (n=1,1%)			1													
Miscellaneous (soft tissue) (n=3, 3%)	1										1					1

DLBCL-Diffuse large B cell lymphoma, MALT - Mucosa associated lymphoid tissue, FL- Follicular lymphoma, MCL- Mantle cell lymphoma, EATL- Enteropathy associated T cell lymphoma, PBL- Plasmablatic lymphoma, BL- Burkitt lymphoma, SMZL- Splenic marginal zone lymphoma, PTCL - Peripheral T cell lymphoma, ALCL- Anaplastic large cell lymphoma, NK cell - Natuiral killer cell lymphoma, TLL- T lymphoblastic lymphoma, BLL- B lymphoblastic lymphoma, NHL- Non hodgkin lymphoma

**Table 2:** Comparison of site and histomorphologic variants of pENL

Author, Year, Place	Total no of NHL (n)	Study period (years)	pENL n (%)	Sites (%)	Histomorphology (%)
Yang et al, 2011, China	5549	8	2986 (53.5)	Waldeyer’s ring (23.7) GIT (22.3) Nose and sinuses (20.4%)	DLBCL (42.5%) ENKTCL (30.9%) ENMZL (11.1%)
Somanath Padhi *et al.*, 2012, India	308	5	68 (22)	CNS (29.5%) GIT (25%)	DLBCL (69%) ENMZL (13.2%)
Mertsoylu H *et al.*, 2014, Southern Turkey	802	10	100 (12.4)	Head and neck (51%) GIT (37%)	DLBCL (53%) ENMZL (13%) FL (7%)
Pai, *et al.*, 2017, South India	114	3	41 (35.96)	GIT (46.34%) Nasopharynx/ Oropharynx (9.75%)	DLBCL NOS B‑cell NHL unclassified
SJ Babu *et al.*, 2018, India	118	4	38 (32.2)	GIT (36.8%) Head and neck (26.3%)	DLBCL (47.4%) B-Cell Lymphoma – Unclassified (21.1%) PTCL (10.5%)
Vasudevan *et al.*, 2022, Kerala, India	2610	5	475 (18.2%)	GIT (24.5%) Head and neck (24.5%)	DLBCL (36.2%) ENMZL (16.1%)
Mishra P *et al.*, 2023, North India	341	5	73 (21.4%)	GIT (31.5%) Head and neck (23.2%)	DLBCL (39.7%) ENMZL (23.2%)
Present study, 2022, India	481	5	109 (22.6)	GIT (39%) Head and neck (26%)	DLBCL (55.1%) FL (9.17%) ENMZL (8.25%)

DLBCL- Diffuse large B cell lymphoma, ENKTCL- Extra nodal NK/T cell lymphoma, ENMZL- Extra nodal marginal zone lymphoma, FL- Follicular lymphoma, CNS- central nervous system, GIT- gastro intestinal tract

**Fig. 6:**
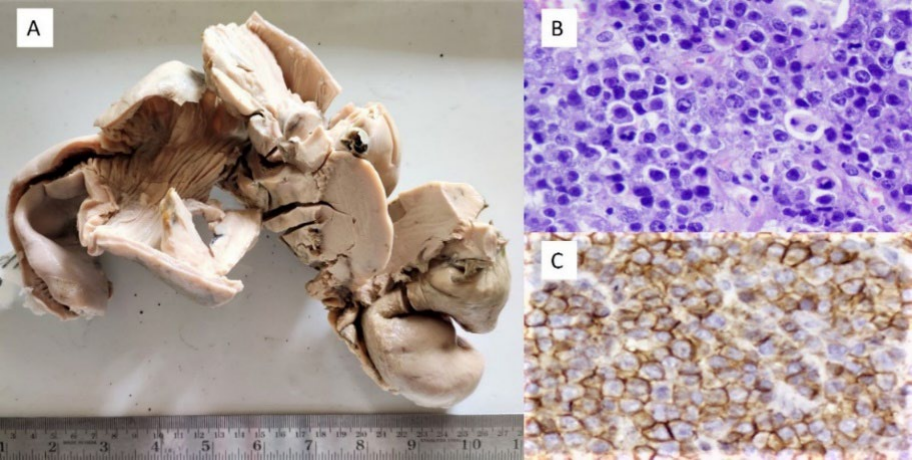
Plasmablastic lymphoma - (A) shows a nodular growth arising from the intestine. (B) H and E x400, Sheets of large cells with plasmacytic differentiation with basophilic cytoplasm. (C) CD138 positivity (CD138, x400).

HIV infection was associated with 12 cases (11%) of pENL with a predominance in males (n=9). DLBCL was the most common subtype (n=7, 58.3%) in pENL associated with HIV. In all cases, 3/109 cases (2.75%) of Plasmablastic lymphoma were seen in HIV-positive patients (Figure 6). Two cases of NHL and NOS with HIV were seen in the colon and stomach. 

In this study, we found 4 pediatric cases of NHL in the age group of 5-16 years. Burkitt lymphoma formed 1.8% (2 cases) and was only seen in the pediatric age group. The sites involved by BL were GIT (Jejunum) and liver. The other histological types occurring in the pediatric age group were ALCL and DLBCL.

**Fig. 7:**
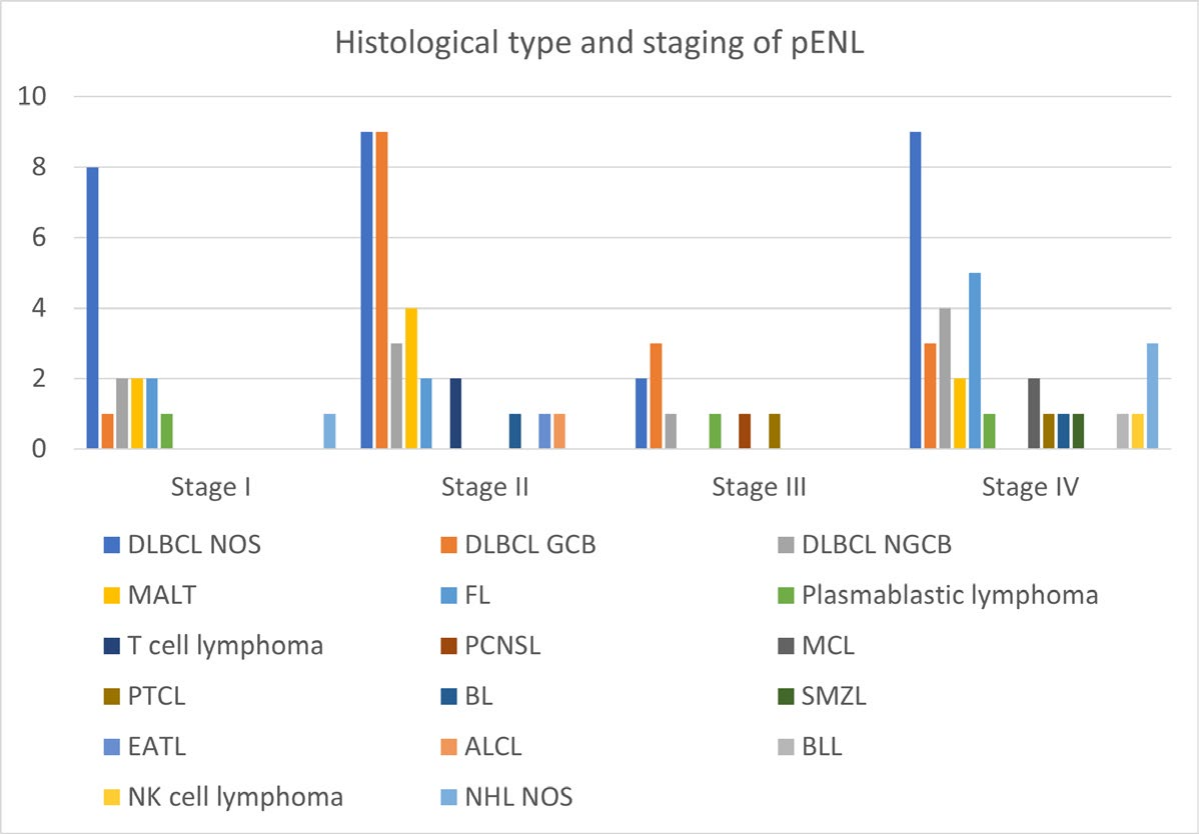
Histological type and staging of pENL (n=109)

pENL were staged according to The Ann Arbor staging system modified by Musshoff and are depicted in Figure 7.

The patients were followed up for a range of 2-36 months. On follow-up (52/109 cases, 47.7%) were lost to follow-up. 2 cases had complete remission, 43(39.4%) cases had stable disease, 1 had progressive disease, 3 had relapsed and 8 died due to complications. As the majority of the cases were lost to follow-up, statistical analysis (OS, DFS) could not be performed.

## Discussion

pENL is a heterogeneous disease in terms of demography, clinical characteristics, etiology, and treatment modalities. Due to the diverse site and morphological types, it is difficult to ascertain the association of specific risk factors. The incidence of ENL varies considerably in different countries like Pakistan, Korea, and China ranging from 45% to 62%(11). In comparison with these data, the incidence in our study (22.6%) is relatively low, which may be because of the shorter duration of study and a smaller number of cases. In this study, males were affected more than females with a M: F ratio of 1.7:1, similar to other studies (12–16). 

Abdominal pain was found to be the most common symptom in studies conducted by Pai *et al. *(11), and SJ Babu *et al. *(17)*.* This is consistent with the results of the current study. The present study revealed that patients with pENL had fewer B symptoms (20%) which is comparable to the findings of other studies (11,14). 

According to our analysis, GIT (39%) is the most common site involved in pENL, which is comparable with several other studies (Table 2). In India, the incidence can vary from one study to another due to the varied geographic and genetic distribution. Various authors have documented CNS as the most common site in pENL (18). In a study by Mehta *et al.* (19), in Rajasthan India, pENL accounted for 54.7% of the cases, with CNS (20.3%) being the commonest site involved. Similarly, a study by Mishra and co-workers (20) documented Head and neck (36%) as the most common site and a series of 2986 cases of pENL study conducted in China (21) demonstrated Waldeyer’s ring as the most commonly involved site. However, our study demonstrates that the small intestine was the most common site involved amongst GI pENL (13.7%), followed by the stomach (11%). This is in line with the findings of Aparna *et al. *(14), and Kajal R Parikh *et al. *(22) who documented the small intestine as the most common site involved in GI pENL. Other studies have stated that the stomach is the most common site involved in GIT (12,23–26). 

Due to strict inclusion criteria, certain authors have excluded Waldeyer’s ring and Tonsil from extranodal sites (18,25). Research from China (21) and Turkey (15) indicates that the head and neck remain the most commonly affected site for pENL. The data suggest that the Nasopharynx (35%) is the most common site affected in the head and neck, followed by the tonsils (27%), and thyroid (18%). Mishra *et al. *(20) described the head and neck (36%) as the predominant pENL site with the tonsil being the commonest site followed by the orbit and nasal cavity. In this particular study, it was found to be the second most common site involved.

In this study, B cell lymphoma constituted 84.4% and T-cell lymphoma of 8.25%, like that of other studies (12,13,15,18). The reason for the increased frequency of the B cell phenotype is due to the clonal expansion of the proliferating cells in the germinal center. T-cell lymphomas arise one-tenth to one-twentieth as often as B-cell lymphomas (27). A study by Vasudevan *et al.* described a high frequency of Adult T-cell leukemia/Lymphoma (ATLL) cases unlike any other studies in India (13). The immunophenotype could not be determined in 8% of the cases. In a series of 810 pENL, multifocal extranodal involvement was seen in 4.2% as demonstrated by Economopoulos, Papageorgiou, Rontogianni *et al.* in a study in Greece (27). 

The most prevalent type of primary gastrointestinal lymphoma (PGIL) is NHL and accounts for 30-40% of cases (28), however, there is evidence of HL secondarily involving the GIT as described by Arora *et al. *(23). DLBCL was the most prevalent histological type, followed by ENMZL in this study like that in literature (22,23,25,26). G. Papaxoinis *et al. *(29) in a series of 128 PGIL described a high proportion of ENMZL (48.4%) followed by DLBCL (44.5%), like a study done in India by Shirsat and Vaiphei *et al. *(24). DLBCL constitutes 25-30% of all lymphomas and represents 40%-70% of gastric lymphomas. The incidence of DLBCL peaked in the 6^th^ decade with M:F ratio of 3.93:1 in the study by Arora *et al. *(23) which was like the current study. The prognosis of PGIL in gastric location, ENMZL phenotype was better than other GI locations and DLBCL phenotype as per the study by Tran *et al.* (26). H. pylori was seen in 7% of PGIL in this study which is much less compared to various other studies (18,24). There is a 6-fold higher risk of developing gastric adenocarcinoma, other NHL, and high-grade transformation in these patients. Successful treatment of H. pylori can result in long-term disease control and regression of the lymphoma (28,30). 

A series of 81 cases of PGIL by Shirsat and Vaiphei *et al.* (24) reported 9 cases of primary intestinal T cell lymphoma with 4 EATL and 5 ALCL cases. The incidence of EATL associated with celiac disease is higher in the West than in the Eastern countries and is rare in Asia (23). We found 2 cases of T cell lymphoma in GIT in this study with 1 case of T cell lymphoma, NOS in the stomach, and 1 case of Enteropathy-associated T cell lymphoma (EATL) in ileum. 

DLBCL (55.1%) was the commonest histological type in the head and neck as seen in the study by Sorrentino *et al.* (31) and Narayana *et al. *(25). FL was the 2nd commonest followed by T-cell lymphoma. Undifferentiated carcinoma is an important differential diagnosis in the nasopharynx which is ruled out by CK. We found one case of NK/T cell lymphoma in the nasopharynx. A strong association of EBV in the pathogenesis of NK/T cell lymphoma was demonstrated in certain studies (25). Sorrentino *et al.* proposed that oral versus nonoral localization of pENL of the head and neck was a significant prognostic indicator and showed a high mortality rate in oral localization (42.42%) of pENL (31). 

FL (9.17%) was the 2nd most common morphologic subtype of pENL in this study. Whereas various studies suggested a low prevalence of FL due to geographic and genetic variation (12,20,32,33). Table 3 gives the comparison of morphologic subtypes in various studies. 

**Table 3:** Comparison of various morphological subtypes in pENL in different studies.

Subtypes	Fujita *et al.*, 2009, Japan (n=395) (%)	H Mertsoylu *et al.*, 2014, Southern Turkey (n=100)	Mehta *et al.*, 2016, India (n=128) (%)	Devi *et al.*, 2018, Northeast India (n=100) (%)
DLBCL	61.2	53	44	45
Follicular lymphoma	5.6	7		5
MALT lymphoma	13.3	13	1	1
Plasmablastic lymphoma			9	
PTCL NOS	1.8		1	
Burkitt lymphoma	1.8		3	6
ALCL	1.5	4	3	15

DLBCL- Diffuse large B cell lymphoma, MALT - Mucosa assocaited lymphoid tissue, PTCL- Peripheral T cell lymphoma, ALCL- Anaplastic large cell lymphoma

According to the literature, primary CNS lymphoma (PCNSL) can be seen in immunocompetent as well as immunocompromised hosts and accounts for 2% of pENL. Immunodeficiency is a well-known risk factor and there is a 3,600-fold increased risk with HIV and EBV infection (30,34). Mehta *et al.* in a study of 128 pENL cases documented 18.6% of PCNSL (19). Our study showed an incidence of 5.5% cases in all immunocompetent patients. 

Primary testicular lymphoma (PTL) forms 9% of testicular neoplasms and 1-2% of all NHL (30). PTLs usually arise in elderly males with bilateral involvement (14), but we reported a case of primary testicular DLBCL in a 27-year-old male with unilateral testicular involvement. It is important to differentiate it from carcinoma/ germ cell tumors as the treatment varies. 

Plasmablastic lymphoma has a very high association with HIV (25,34). It was initially described in the oral cavity, other sites of involvement as per the literature are the testis and rectum (25). Various studies have described a predilection of PBL for the oral cavity in HIV-positive patients (23,34). In this study we had 3 cases of PBL associated with HIV and the sites involved were the rectum, eye, and nasal cavity.

In HIV-associated pENL, GIT was the common site involved with DLBCL being the most common histological type, followed by PBL. These data were like the other studies (23,35). Studies have shown that patients with concurrent HAART therapy had better overall survival than those who were not on treatment (34,35). 

According to a population-based study by Gupta *et al.* (36), the primary site of extranodal DLBCL has a significant role in the prognosis and outcome of patients. CNS relapse, primary EN-DLBCL of the nervous system, respiratory system, pancreas, and hepatobiliary involvement had worse outcomes. Patients with primary DLBCL of the bone marrow had poorer survival (16). A study by Lal *et al. *(37) stated that in patients with pENL presented at an early stage, the prognosis and overall survival largely depend on the IPI score and not the site of pENL. 

It is worth noting that this study has certain limitations that should be considered when interpreting the results. Specifically, almost half (47.7%) of the patients who participated in the study were lost to follow-up, a proportion that may impact the generalizability of the results. Due to this, it was not possible to conduct statistical analysis (OS) on the data, which could have provided valuable insights into the outcomes of the study. As such, it is important to bear in mind that the conclusions drawn from this study may not be fully representative of the entire patient population.

## Conclusion

This retrospective study illustrates the varied pattern of distribution of common and rarer pENL in a tertiary care center. The results are comparable with other studies in the literature. A diagnosis of pENL requires the exclusion of secondary involvement of extranodal sites by a primary nodal disease. The clinical presentation depends upon the site of involvement. Immunohistochemistry (IHC) is crucial in subclassifying the lymphoma and excluding other histological mimickers such as Poorly differentiated carcinoma or Small round cell tumors. Further studies with adequate follow-up and response to treatment are the need of the hour for a better understanding of pENL.
